# The effects of tualang honey on female reproductive organs, tibia bone and hormonal profile in ovariectomised rats - animal model for menopause

**DOI:** 10.1186/1472-6882-10-82

**Published:** 2010-12-31

**Authors:** Siti SM Zaid, Siti A Sulaiman, Kuttulebbai NM Sirajudeen, Nor H Othman

**Affiliations:** 1 Department of Environmental Sciences, Faculty of Environmental Sciences, Universiti Putra Malaysia, 43400 UPM Serdang, Selangor, Malaysia; 2 Department of Pharmacology, School of Medical Sciences, Health Campus, Universiti Sains Malaysia, 16150 Kubang Kerian, Kelantan, Malaysia; 3 Department of Chemical Pathology, School of Medical Sciences, Health Campus, Universiti Sains Malaysia, 16150 Kubang Kerian, Kelantan, Malaysia; 4 Department of Pathology, School of Medical Sciences, Health Campus, Universiti Sains Malaysia, 16150 Kubang Kerian, Kelantan, Malaysia

## Abstract

**Background:**

Honey is a highly nutritional natural product that has been widely used in folk medicine for a number of therapeutic purposes. We evaluated whether Malaysian Tualang honey (AgroMas, Malaysia) was effective in reducing menopausal syndrome in ovariectomised female rats; an animal model for menopause.

**Methods:**

The rats were divided into two control groups and three test groups. The control groups were sham-operated (SH) and ovariectomised (OVX) rats. The SH and OVX control rats were fed on 0.5 ml of distill water. The rats in the test groups were fed with low dose 0.2 g/kg (THL), medium dose, 1.0 g/kg (THM) and high dose 2.0 g/kg (THH) of Tualang honey in 0.5 ml of distill water. The administration was given by oral gavage once daily for 2 weeks. The reproductive organs (uterus and vagina), tibia bone and aorta were taken for histopathological examination while serum for hormonal assays.

**Results:**

Administration of Tualang honey for 2 weeks to ovariectomised rats significantly increased the weight of the uterus and the thickness of vaginal epithelium, restored the morphology of the tibia bones and reduced the body weight compared to rats in the ovariectomised group. The levels of estradiol and progesterone, in honey treated groups were markedly lower than that in the OVX group. At low doses (0.2 g/kg; THL group) of Tualang honey there was an increased in serum free testosterone levels compared to OVX group (P < 0.01). Progesterone concentrations was significantly decreased in the OVX group as compared to SHAM group (P < 0.05).

**Conclusions:**

Tualang honey was shown to have beneficial effects on menopausal (ovariectomised) rats by preventing uterine atrophy, increased bone density and suppression of increased body weight. Honey could be an alternative to HRT.

## Background

Menopause is the time of life in women when menstrual cycles and reproductive function permanently cease due to loss of ovarian follicular activity [1,2]. Clinically, natural menopause can be diagnosed in women in their 40s or 50s after 12 consecutive months of amenorrhea for which no other obvious pathological or physiological causes can be found [3,4]. It could also be induced by surgery, chemotherapy or radiation [1,5].

There are various early and late complications of menopausal period frequently referred as postmenopausal syndrome such as vasomotor, psychogenic and sexual problems. Later onset include increase incidence of atherosclerosis, ischemic heart disease, stroke and osteoporosis [1,6,7]. In order to reduce these complications, hormone replacement therapy (HRT) has been prescribed extensively over the last 25 years [8,9]. Long-term HRT use increases the risk of breast cancer, endometrial cancer, thromboembolic events and vaginal bleeding [10]. Such findings have resulted in a search for alternatives to HRT.

Honey is a natural product that has been widely used for its therapeutic effect. It has been reported to contain about 200 substances such as mixture of sugars (fructose, glucose, maltose and sucrose), small amounts of other constituents such as minerals, proteins, vitamins, organic acids, flavonoids, phenolic acids, enzymes and other phytochemicals [11]. Tualang honey is wild multi-floral honey found in the Malaysian Rain Forest in Malaysia. The honey bee specie is *Apis Dorsata*. Study on postmenopausal women taking Tualang honey at 20 mg/day for 4 months were found to have similar bone densitometry findings when compared with hormone replacement therapy [12].

Royal jelly and propolis are other beehive products that have been scientifically proven for their improvement of menopausal syndrome [13,14]. Thus far, there are no data on the benefits of honey on menopausal syndrome. We conducted controlled experimentation using ovariectomised rats as an animal model for menopausal syndrome to study the effect of Malaysian Tualang honey on body weights, reproductive organs, tibia bone, aorta and assayed the hormonal profiles of these rats.

## Methods

The study was done on 3 months old, thirty-five virgin female Sprague Dawley rats. The weights of the rats were about 220-240 g and they were obtained from the Laboratory Animal Research Unit (LARU), Health Campus, Universiti Sains Malaysia. After acclimatization for a week, the rats were divided into 5 groups (n = 7); 2 control and 3 test groups. The control rats either had bilateral ovariectomy (OVX group) or sham-operated (SHAM group) under diethyl ether anesthesia. The rats in the test group had bilateral ovariectomy. The control rats (SHAM and OVX) were given 0.5 ml of distilled water. The test rats were given low dose; 0.2 g/kg (THL group), medium dose; 1.0 g/kg (THM group) and high dose; 2.0 g/kg (THH group) of Tualang honey topped up to 0.5 ml of distill water. The administration was started one day after surgery. The test and control rats were oral gavaged with 0.5 ml honey or water at approximately 9:00-10:00 am everyday for 2 weeks. Throughout the treatment period all rats had daily vaginal smear cytology to determine the estrous cycle (reproductive cycle). Daily measurement of body weight and the total food intake was recorded.

The Tualang honey used was supplied by Federal Agricultural Marketing Authority (FAMA), Ministry of Agriculture and Agro-Based Industry, Malaysia. The water concentration of the honey was standardized by FAMA at 18%.

After two weeks, the rats were sacrificed under excess diethyl ether anesthesia. The blood samples were taken from abdominal aorta and centrifuged at 2500 rpm for 15 min at 4°C to extract the serum for hormonal profile analysis. The reproductive organs (uterine horn and vagina) were carefully removed, cleaned, weighed and fixed in 10% formalin. The heart and the attached aorta were dissected and immersed in 10% formalin without flushing out the blood. The right tibia was removed from each rat and fixed with 70% ethanol. The mentioned organs were stained with Hematoxylin and Eosin (H&E) and examined under light microscopy at 20× magnification. The pathologist who evaluated the histology of these organs (NHO - the corresponding author) was blind to the treatment the rats received. The mean vaginal epithelium and endometrial uterine thicknesses was calculated based on measurements in six randomly chosen areas of the sections using a computer-aided program (Image-Pro Plus Version 5.0, USA).

Data analysis was done using Statistical Package for Social Sciences (SPSS version 12.0.1) software. Statistical analysis was performed using the Kruskall-Wallis test for multiple comparisons, followed by Mann-Whitney U-test to compare differences between two groups. Results were expressed as median and interquartile range (IR: the difference between the 75 and 25 percentile). P values of less than 0.05 were considered statistically significant.

The protocol used in this study was approved by the Animal Ethics Committee, Universiti Sains Malaysia (approval # PPSG/07(A)/044).

## Results

The relative weights of uterus, vagina and tibia bone of control and test rats are shown in Table 1. The relative weights of uterus showed marked decrease after ovariectomy. On administration of Tualang honey at 0.2 g/kg (THL group) and 1.0 g/kg (THM group) for 2 weeks after ovariectomy, there was significant prevention (P < 0.05) of the loss of uterus relative weight compared to non-honey treated rats (OVX group). Similarly, in honey-treated rats, the weights of the vagina also showed significant increased values when compared to rats in OVX group. The thickness of the vaginal epithelium and endometrium is also significantly different in honey-treated rats compared to OVX group (Table 2).

**Table 1:** Effects of Tualang honey on relative organ weight (mg/g body weight) after 2 weeks administration

	SHAM	OVX	THL (0.2 g/kg)	THM (1.0 g/kg)	THH (2.0g/kg)
Vagina	60.51 (13.00) **	48.26 (4.00)	54.98 (5.00)*	52.68 (3.00)*	55.49 (9.00)*
Uterus	178.38 (113.00) **	62.35 (21.00)	83.49 (15.00)*	86.16 (20.00)*	69.46 (16.00)
Tibia	232.20 (32.00)	215.15 (16.00)	220.79 (12.00)	220.47 (11.00)	219.25 (27.00)

SHAM = Sham-operated control rats; OVX = ovariectomised non-honey treated control rats

THL = Tualang Honey, low dose; THM = Tualang Honey, medium dose; THH = Tualang Honey, high dose

All values are expressed as the median (IR).

^a ^Kruskal - Wallis test.

*P < 0.05 and **P < 0.01 vs. OVX (Mann-Whitney U - test)

**Table 2:** Effects of Tualang honey on epithelial vaginal thickness and endometrial uterine thickness after 2 weeks administration

	SHAM	OVX	THL (0.2 g/kg)	THM (1.0 g/kg)	THH (2.0 g/kg)
Vaginal epithelium (μm)	49.72 (6.00)**	28.9 (7.00)	50.15 (19.00)**	43.41 (21.00)*	38.65 (9.00)**
Endometrium (μm)	436.05 (96.00)**	78.39 (168.00)	517.35 (125.00)*	489.09 (90.00)	506.81 (120.00)

SHAM = Sham-operated Control rats; OVX = overictomised non-honey treated Control rats

THL = Tualang Honey, low dose; THM = Tualang Honey, medium dose; THH = Tualang Honey, high dose

All values are expressed as the median (IR).

^a ^Kruskal - Wallis test.

*P < 0.05 and **P < 0.01 vs. OVX (Mann-Whitney U - test)

**Figure 1:**
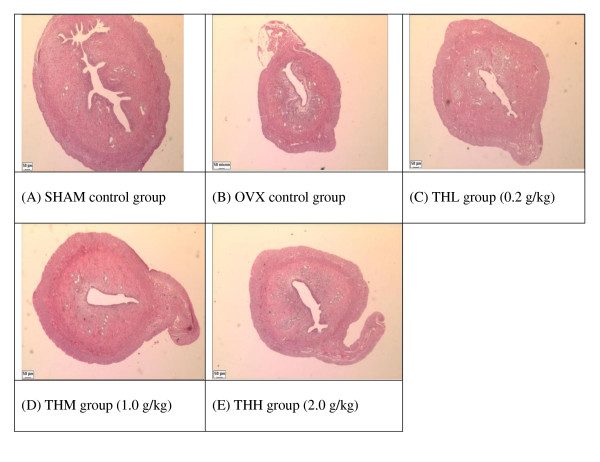
**Effects of Tualang honey on uterine morphology in rats**. OVX control group showed an atrophic uterus with cuboidal endometrial epithelium, endometrium with cuboidal inactive cells and stroma in less cellular. After 2 weeks administration of Tualang honey at all doses has resulted in a partially reversal of uterine atrophy. The increased thickness of endometrium in all treated groups was due to an increased in the amount of collagen in the stroma. All pictures are stained with H&E and examined under ×20 magnification: SHAM = Sham-operated control rats, OVX = ovariectomised non-honey treated control rats, THL = Tualang Honey, low dose, THM = Tualang Honey, medium dose, THH = Tualang Honey, high dose

**Figure 2:**
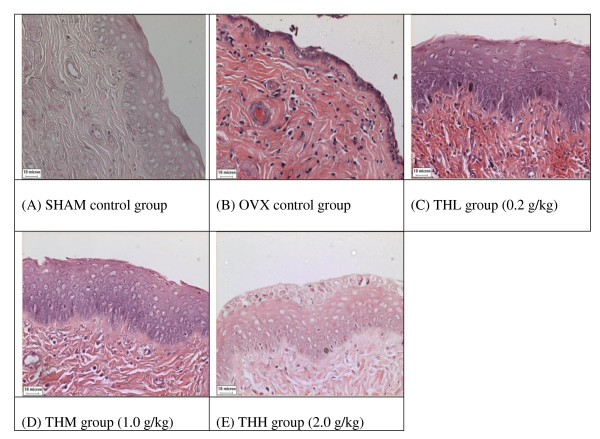
**Effects of Tualang honey on vagina morphology in rats**. OVX control group showed an atrophic vaginal epithelium with composed of a few layers of flattened cells. After 2 weeks administration of Tualang honey at all doses caused a complete reversal of the vaginal atrophy. This effect was accompanied by hyperplasia and hypertrophic of vaginal epithelium. The cytoplasmic vacuolization was clearly present in THH group. All pictures are stained with H&E and examined under ×20 magnification: SHAM = Sham-operated control rats, OVX = ovariectomised non-honey treated control rats, THL = Tualang Honey, low dose, THM = Tualang Honey, medium dose, THH = Tualang Honey, high dose

Under light microscopy, we observed marked atrophy of the rat uterus to about half its former size once the rats are ovariectomised (OVX group). The size increased to near normal in honey-treated rats (Figure 1). Likewise the endometrial thickness showed atrophy in the OVX group. After 2 weeks administration of Tualang honey at all doses caused a complete reversal of the vaginal atrophy (Figure 2). This effect was accompanied by mild hyperplasia of vaginal epithelium. Cytoplasmic vacuolization of vaginal epithelium was noted in rats on high dose of 2.0 g/kg Tualang honey (THH group - Figure 2E). The general histological features of the squamous epithelium of the vagina of the honey-treated rats closely resembled to that in SHAM rats.

**Figure 3:**
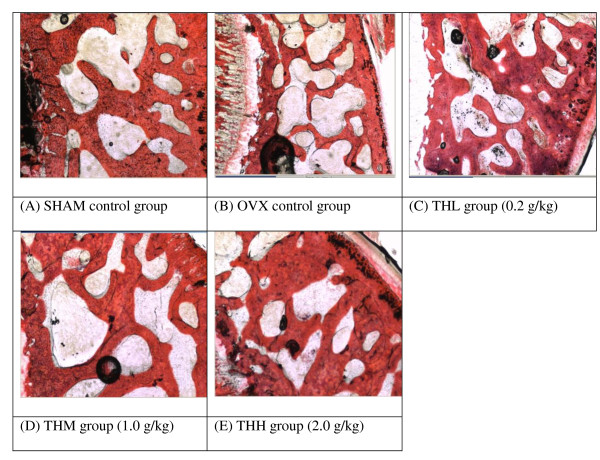
**Effects of Tualang honey on trabecular thickness of tibia bone**. Histopathological examination (HPE) indicated that Tualang honey is able to restore the trabecular thickness in tibia bone of ovariectomised rats. All pictures are stained with H&E and examined under ×20 magnification: SHAM = Sham-operated control rats, OVX = ovariectomised non-honey treated control rats, THL = Tualang Honey, low dose, THM = Tualang Honey, medium dose, THH = Tualang Honey, high dose

The tibia bone weights was also heavier in honey-treated rats, however they are not statistically significant (Table 1). Histological examination showed, Tualang honey was able to restore the morphology of tibia bone of ovariectomised rats to nearly its non-ovariectomised state (Figure 3).

The effects of Tualang honey on serum estradiol, progesterone, free testosterone and FSH (follicle stimulating hormone) are shown in Table 3. No significant changes in serum concentrations of estradiol and FSH was noted among all groups, however the values of these parameters in all honey treated groups was markedly lower than that in the OVX group. At low doses (0.2 g/kg; THL group) of Tualang honey there was a increased in serum free testosterone in the ovariectomised rats (P < 0.01). Progesterone concentrations was significantly decreased in the OVX group as compared to SHAM group (P < 0.05) but no significant changes was observed in Tualang honey treated rats. The values of progesterone in the THL and THH groups were markedly higher than that in the OVX group.

**Table 3:** Effects of Tualang honey on serum estradiol, progesterone, free testosterone, follicle-stimulating hormone (FSH) and luteinizing hormone (LH) after 2 weeks administration

	SHAM	OVX	THL (0.2 g/kg)	THM (1.0 g/kg)	THH (2.0 g/kg)
Estradiol (pg/ml)	14.82 (13.00)	12.58 (9.00)	7.42 (6.00)	7.55 (8.00)	5.21 (0.01)
Progesterone (ng/mg)	14.59 (13.00)*	4.28 (4.00)	6.18 (14.00)	3.72 (4.00)	8.92 (11.00)
Free testosterone (pg/ml)	0.27 (0.01)**	0.03 (0.01)	0.05 (0.01)*	0.04 (0.01)	0.05 (0.10)
FSH (mlU/ml)	0.53 (0.01)	0.63 (0.01)	0.43 (1)	0.61 (0.01)	0.4 (0.01)
LH (mlU/ml)	ND^a^	ND	ND	ND	ND

SHAM = Sham-operated Control rats; OVX = overictomised non-honey treated Control rats

THL = Tualang Honey, low dose; THM = Tualang Honey, medium dose; THH = Tualang Honey, high dose

All values are expressed as the median (IR).

^a ^Kruskal - Wallis test.

*P < 0.05 and **P < 0.01 vs. OVX (Mann-Whitney U - test)

^a^Not detectable (values below detection limit).

The changes in body and total food intake in all groups are shown in Table 4. The body weights of ovariectomised control rats were significantly greater than the SHAM control group (P < 0.05). The ovariectomy-induced increase in body weights were significantly abolished after administration of Tualang honey at all doses. The total food intakes were significantly higher in OVX group as compared to SHAM and honey treated groups. All animals in SHAM group demonstrated regular 4 to 5 days of estrous cycle. Control ovariectomised and all honey treated rats showed metestrus and diestrus phase.

**Table 4:** Effects of Tualang honey on changes in body weight and total food intake after 2 weeks administration

	SHAM	OVX	THL (0.2 g/kg)	THM (1.0 g/kg)	THH (2.0 g/kg)
Body weight on the first day of treatment (g)	229.0	226.0	221.0	229.0	229.0
Body weight at sacrifice (g)	234.0	261.0	252.0	259.0	258.0
Changes in body weight (%)	4.63 (3.00)**	17.60 (5.00)	15.38 (3.00)*	13.51 (7.00)*	10.73 (8.00)**
Total food intake (g)	211.0 (70.00)	269.0 (58.00)	196.0 (7.00) **	218.0 (36.00)*	219.0 (18.00)*

SHAM = Sham-operated Control rats; OVX = overictomised non-honey treated Control rats

THL = Tualang Honey, low dose; THM = Tualang Honey, medium dose; THH = Tualang Honey, high dose

All values are expressed as the median (IR).

^a ^Kruskal - Wallis test.

*P < 0.05 and **P < 0.01 vs. OVX (Mann-Whitney U - test)

Histological examination of the aorta did not show atheroma formation at the endothelial wall of control and honey-treated rats.

## Discussion

Our study showed administration of Tualang honey to ovariectomised rats for 2 weeks significantly increased the weights of the uterus, increased the thickness of vaginal epithelium, restore the tibia bones and reduced the body weights compared to control non-honey treated rats. It is interesting to note that significant change in epithelial thickness, vagina and uterus weights were seen in rats with lower doses of honey. The tibia bone weights were also heavier in lower dose compared to higher doses of honey, though not statistically significant.

The animal model we used is akin to women in menopause. Vaginal smear of all animals in SHAM control group in our study demonstrated regular 4 to 5 days of estrous cycle while control ovariectomised and honey treated groups showed metestrus or diestrus phases only. The result of these estrus cycle follow a predictable cyclic pattern in a succinct 4-day time period. The 4-day estrus cycle is compromised of 1-day phase and can be divided into diestrus 1, diestrus 2, proestrus and estrus cycle [15]. In SHAM control group, the existence of proestrus phase is analogous to the late follicular/early luteal phases in fertile woman, which is characterized by a rapid rise and fall of serum estradiol that immediately precedes ovulation (estrus). In ovariectomised and honey treated rats we noted only metestrus or diestrus phase indicating no ovulation had occurred analogous to menopausal women. According to these similarities with human, ovariecomised animals have proven to be useful in studying various aspects of hormone-induced changes [16].

Ovariectomy is a surgical procedure to induce menopause in experimental animals. We showed in this study the rats which were ovariectomised and not treated with honey had a dramatic decrease in the weights of the reproductive organs particularly uterus and vagina compared with rats which underwent only sham operation. The changes in weights seen in ovariectomised rats were due to atrophy of endometrium and vaginal epithelium resulting from lack of hormones secreted by the ovaries. These imply that these tissues/organs are affected in menopause. Menopause also reduces the ability of epithelial cells to produce glycogen which maintains low pH in vaginal fluid [15]. Changes in the vaginal epithelium may lead to disturbances in vaginal sensation noted after menopause.

On administration of Tualang honey at all doses, we noted there was significant lack of atrophy of the vaginal epithelium. The uterine endometrium maintained the thickness in non-ovariectomised rats. We also noted vacuolation of the vaginal epithelial cells implying increase in mucopolysacharide (glycogen) content. If inferred to human, such finding may be of benefit to women who experience vaginal dryness after menopause. The positive effects of Tualang honey is probably due to its high nutritional contents, particularly flavonoids [17,18]. Flavonoids particularly kaempferol and quercetin have been shown to have weak estrogenic activity [16,17].

To date, there is no published scientific evidence on the benefits of honey on reproductive organs however the effects of other beehive products namely propolis and royal jelly have been reported [13,14]. The compositions of honey and beehive products are considerably similar but qualitatively and quantitatively are dissimilar depending on the regional plant ecology [18,19]. The improvement of uterus and vagina atrophy is due to the presence of biologically active estrogen-like molecules or phytoestrogens in honey. Further, flavonoid, which is present in honey are antioxidant compounds which could retard biologically destructive chemical reactions in living organisms through their ability to scavenge oxidants and free radicals [20-22].

We observed the morphology of tibial bones of the ovariectomised rats treated with Tualang were like those of control non-ovariectomised rats. The positive effect on bone density is probably due to honey containing high flavonoids and gluconic acid. Previous investigators claimed that gluconic acid which is the major organic acid in honey and carbohydrate constituents could enhance calcium absorption in the bone of rats [23,24]. Kaempferol (flavonoids compounds in honey) also has osteogenic effect in ovariectomized rats [25].

Ovariectomy can induce an increase in body weight and food intake [6,26]. Such findings were also observed in the rats of our study. Previous studies have shown that estrogen plays an important role in lipid metabolism during premenopausal years [27]. Estrogen insufficiency during menopause is thought to be largely responsible for an increase in adiposity, particularly abdominal fat accumulation. When postmenopausal women receive estrogen replacement therapy they do not display the characteristic pattern of abdominal weight gain associated with menopause. Changes in androgenic profile nearer to that of man, have also been suggested to be responsible for changes in body fat distribution during this period [1].

We observed body weight gain was significantly prevented by Tualang honey administration at all doses. This could be due to the nutrients found in honey; anti-oxidants such as flavonoids and phenolic acids; enzymes such as glucose oxidase, invertase, diastase (amylase), catalase and acid phosphatase; organic acids such as gluconic acid, caffeic, ferulic, butyric, acetic, formic, lactic, succinic, malic, citric, maleic, oxalic and pyroglutamic and other nutriens such as vitamins and minerals [18]. Certain bioactive compounds in Tualang honey might have prevented the gain in weight of ovariectomised rats. We also observed the total food intake in ovariectomised Tualang honey-treated rats were significantly lower than ovariectomised non-honey treated rats. The explanation could be due to honey contains a high concentrated source of energy (313 calories per 100 g) which help in reducing food intake.

The hormonal profiles in our honey treated rats show mixed findings. At low doses (0.2 g/kg; THL group) of Tualang honey there was an increased in serum free testosterone levels compared to OVX group (P < 0.01). The level of estradiol reduces on treatment with honey although not dose-related. The level of progestogens is increased in low and high dose but not in medium dose of honey. The reason for the mixed findings could be due to our rats was only exposed to two weeks of honey. It would be interesting to see the hormonal changes if they are exposed for a longer period.

The rats in our study did not develop atheroma perhaps also because of short study period. Previous studies have reported that rats do not develop spontaneous atherosclerosis except after 3 to 6 months on high fat and cholesterol diet modifications [28,29].

## Conclusions

Daily consumption of Tualang honey for 2 weeks in female adult ovariectomised rats, a model for menopausal symptoms, provided protective and beneficial effects. Tualang honey was shown to prevent uterine atrophy, vaginal epithelium atrophy, promote increased bone density and suppress the increased of body weight seen in menopausal state. Clinical trials are required to see if these benefits could be translated to post menopausal women.

## Competing interests

The authors declare that they have no competing interests.

## Authors' contributions

SMZ (1^st ^author) carried out all the lab work, the analysis of the data and drafted the manuscript. SBS (2^nd ^author) participated in the design of the study. KNS (3^rd ^author) was involved in the hormonal analysis. NHO (corresponding author) was involved in the study design, examined the histology of the organs, drafted and finalised the manuscript. All authors read and approved the manuscript before submission.

## Pre-publication history

The pre-publication history for this paper can be accessed here:

http://www.biomedcentral.com/1472-6882/10/82/prepub
